# Supplementary material for: HSSM: A Widely Applicable Toolbox for Hierarchical Bayesian Neurocognitive Modeling
Source: bioRxiv. 2026 Jun 9:2026.06.05.730398. Preprint. [Version 1] doi: 10.64898/2026.06.05.730398 (PMC13277915; doi:10.64898/2026.06.05.730398)
Supplement: Supplementary file 1 [file NIHPP2026.06.05.730398v1-supplement-1.pdf]

bioRxiv preprint doi: https://doi.org/10.64898/2026.06.05.730398; this version posted June 9, 2026. The copyright holder for this preprint (which was not certified by peer review) is the author/funder, who has granted bioRxiv a license to display the preprint in perpetuity. It is made available under a CC-BY 4.0 International license.

Supplementary figures

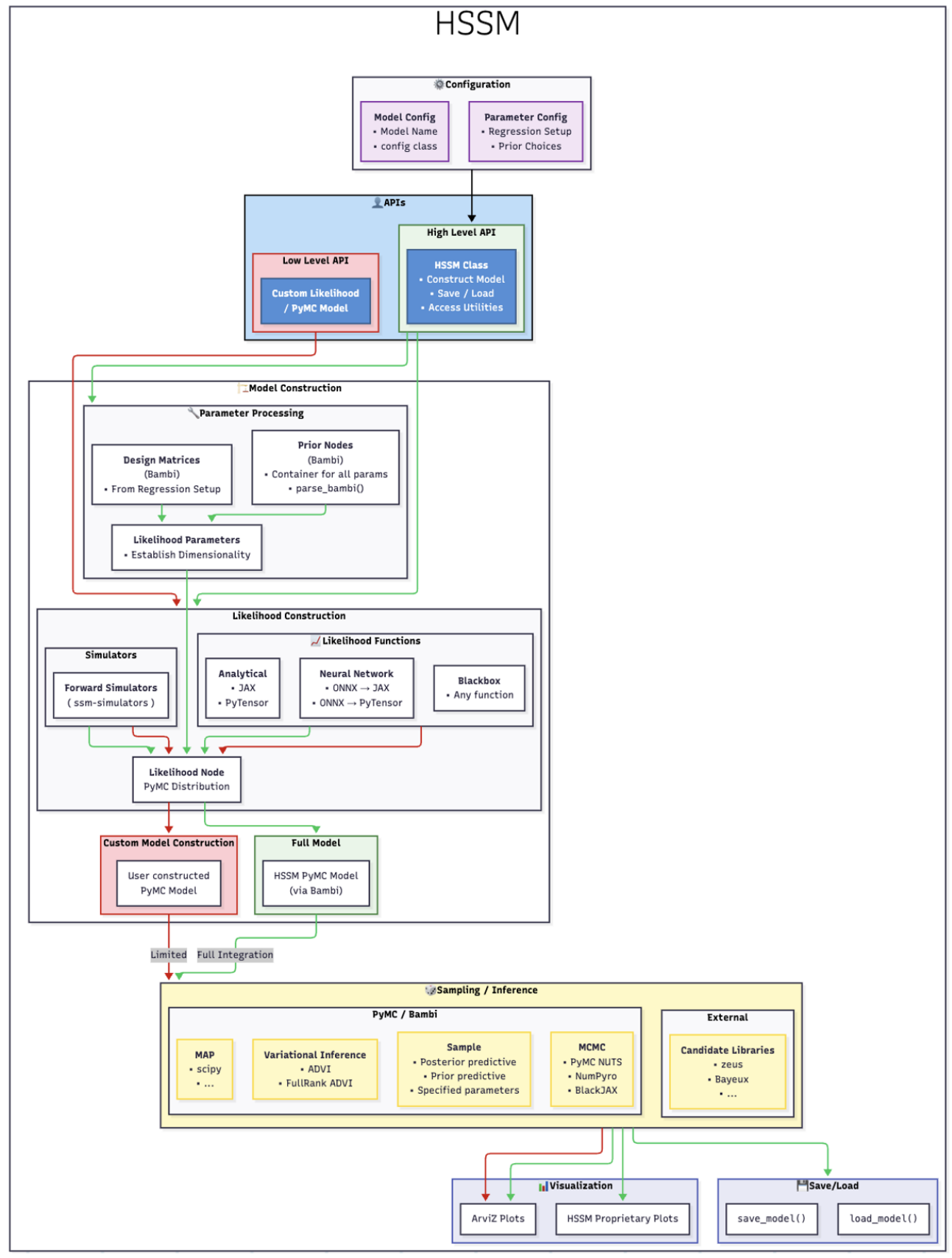

Figure 3—figure supplement 1. A more complete view on the architecture of the `HSSM` package. HSSM serves as the user-facing hub of the ecosystem and is designed around two complementary APIs (blue block). The high-level API centers on the `HSSM` class and consumes model and parameter configurations — including regression setup and optionally prior choices — to construct a full hierarchical Bayesian model automatically. The low-level API exposes the underlying pre-assembled PyMC distributions, allowing advanced users to build entirely custom PyMC models while retaining access to HSSM's cognitive process model likelihoods. The likelihood layer accommodates multiple sources — analytical closed-form expressions, neural network surrogates loaded from ONNX (via either JAX or PyTensor), and blackbox user-provided functions — alongside forward simulators drawn from `ssm-simulators` for prior and posterior predictive sampling (users can provide their own if desired). The assembled likelihood node is a standard PyMC distribution and can be fit with a wide range of inference backends (yellow block): gradient-based MCMC via PyMC NUTS, NumPyro, or BlackJax; variational inference (ADVI, FullRank ADVI); MAP estimation; or external samplers. Outputs are compatible with ArviZ diagnostics and HSSM's proprietary plotting utilities, and models can be persisted via native save/load methods.

bioRxiv preprint doi: https://doi.org/10.64898/2026.06.05.730398; this version posted June 9, 2026. The copyright holder for this preprint (which was not certified by peer review) is the author/funder, who has granted bioRxiv a license to display the preprint in perpetuity. It is made available under a CC-BY 4.0 International license.

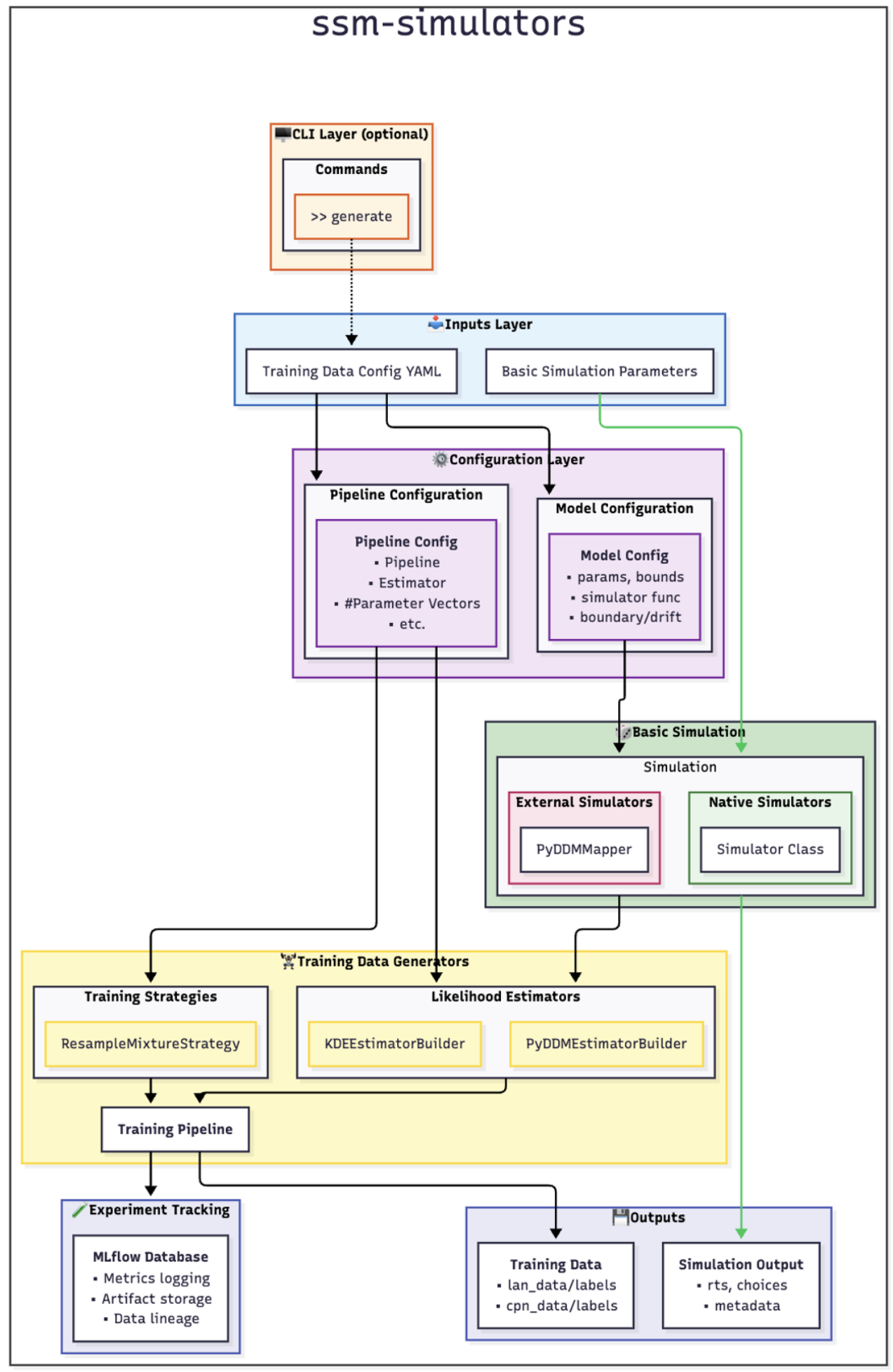

Figure 8—figure supplement 1. Architecture of the `ssm-simulators` package. The package provides two core functionalities: forward simulation of cognitive process models via the `Simulator` class, and a modular training data generation pipeline that combines configurable likelihood estimators and sampling strategies to produce structured datasets for downstream likelihood approximation. The package has a modular organization across level of functionality. Simulators can be constructed natively via `ssm-simulators` or e.g. via the `PyDDM` backend (***Shinn et al., 2020***) (with a vision toward extension). Training data generators are designed to combine different types of likelihood estimators as training signals for downstream networks (currently implemented are likelihoods based on `PyDDM` (***Shinn et al., 2020***) and our native approach following (***Fengler et al., 2021***).

bioRxiv preprint doi: https://doi.org/10.64898/2026.06.05.730398; this version posted June 9, 2026. The copyright holder for this preprint (which was not certified by peer review) is the author/funder, who has granted bioRxiv a license to display the preprint in perpetuity. It is made available under a CC-BY 4.0 International license.

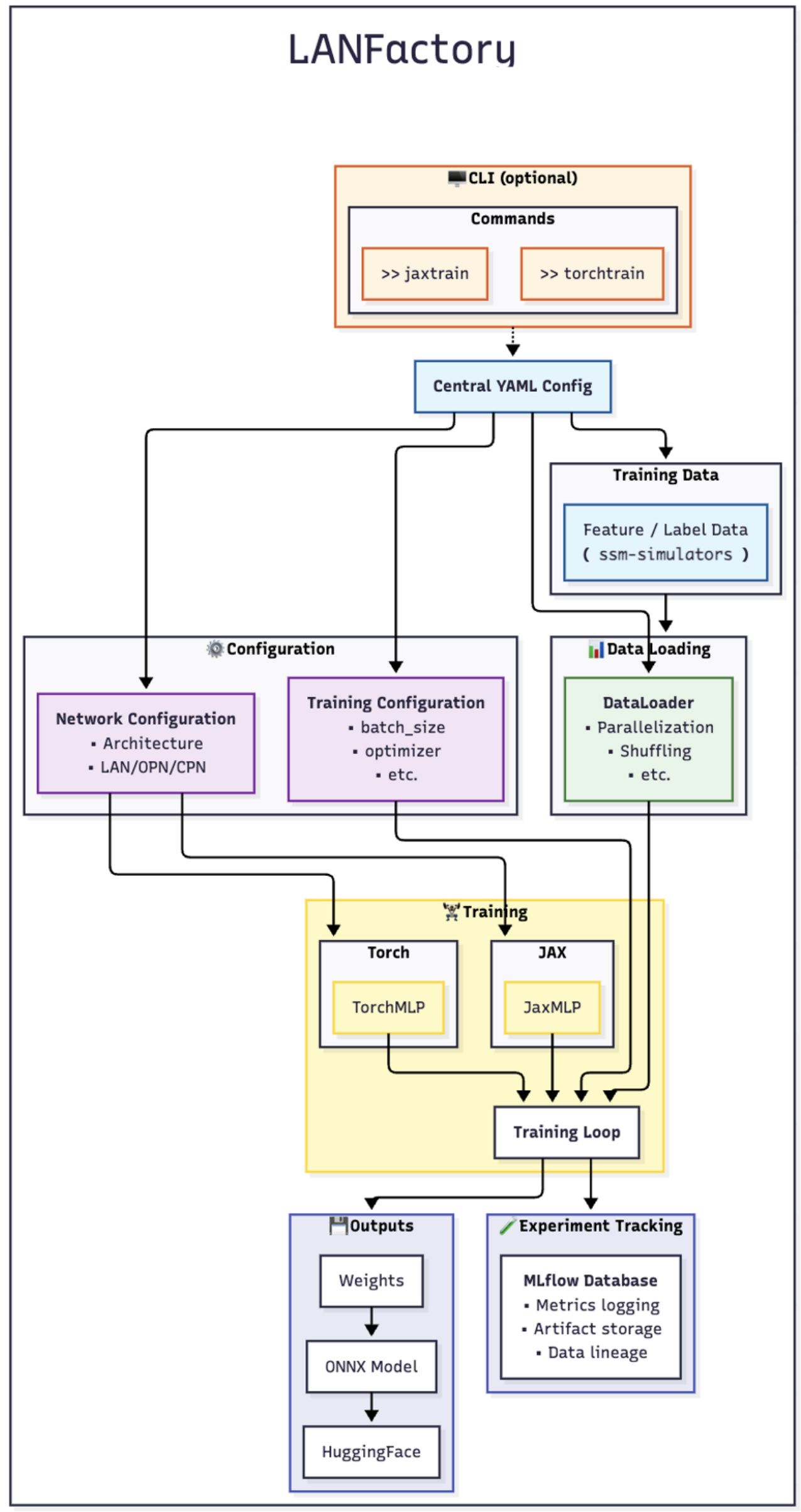

Figure 9—figure supplement 1. Detailed architecture of the `LANFactory` package. The package is configured through a central YAML file, optionally invoked via the `jaxtrain` and `torchtrain` CLI commands. The configuration drives three parallel substructures: network configuration (architecture choice and target network type — LAN, OPN, or CPN), training configuration (batch size, optimizer, and other hyperparameters), and data loading (parallelized, shuffled feature/label data, typically produced by `ssm-simulators`). The training subsystem provides parallel JAX and PyTorch implementations via the `JaxMLP` and `TorchMLP` class abstractions, both feeding into a unified training loop. Trained weights are exported to ONNX and uploaded to HuggingFace for downstream consumption by `HSSM` or other tools. Throughout training, an optional MLflow integration logs metrics, stores artifacts, and tracks data lineage to support reproducible network development.
